# Analyses of seven new whole genome sequences of cassava brown streak viruses in Mozambique reveals two distinct clades: evidence for new species

**DOI:** 10.1111/ppa.13001

**Published:** 2019-03-10

**Authors:** J. J. G. Amisse, J. Ndunguru, F. Tairo, E. Ateka, L. M. Boykin, M. A. Kehoe, N. Cossa, C. Rey, P. Sseruwagi

**Affiliations:** ^1^ Mozambique Agricultural Research Institute Posto Agronómico de Nampula, PO Box 622, Rua de corrane, km 7 Nampula Mozambique; ^2^ Jomo Kenyatta University of Agriculture and Technology PO Box 62000, City Square Nairobi Thika Kenya; ^3^ Tanzania Agricultural Research Institute – Mikocheni PO Box 6226 Dar es Salaam Tanzania; ^4^ The University of Western Australia ARC Centre of Excellence in Plant Energy Biology and School of Molecular Sciences Crawley 6009 Australia; ^5^ Department of Primary Industries and Regional Development Diagnostic Laboratory Service Locked Bag 4 Bentley Delivery Centre South Perth 6983 Australia; ^6^ School of Molecular and Cell Biology University of the Witwatersrand 1 Jan Smuts Ave, Braamfontein Johannesburg 2000 South Africa

**Keywords:** *Cassava brown streak virus*, complete genome sequence, genetic diversity, Mozambique

## Abstract

Cassava brown streak disease (CBSD) caused by *Cassava brown streak virus* (CBSV) and *Uganda cassava brown streak virus* (UCBSV) is a major constraint to cassava production in Mozambique. Full genome sequences of CBSD‐associated virus isolates contribute to the understanding of genetic diversity and the development of new diagnostic primers that can be used for early detection of the viruses for sustainable disease management. This study determined seven new whole CBSV genomes from total RNA isolated from cassava leaves with CBSD symptoms collected from Nampula and Zambezia in Mozambique. Phylogenetic analyses of the new genomes with published CBSV and UCBSV sequences in GenBank grouped the CBSV isolates from Mozambique into two distinct clades together with CBSV isolates from Tanzania. Clade 1 and 2 isolates shared low nucleotide (79.1–80.4%) and amino acid (86.5–88.2%) sequence identity. Further, comparisons within the seven new CBSV isolates, and between them and the single published complete CBSV sequence (CBSV_MO_83_FN434436) from Mozambique, revealed nucleotide sequence identities of 79.3–100% and 79.3–98%, respectively, and amino acid identities of 86.7–100% and 86.7–98.8%. In addition, using RDP4, a recombination analysis comprising all CBSV and UCBSV genome sequences from GenBank detect 11 recombination events. Using several comprehensive evolutionary models and statistical programs, it was confirmed that CBSV and UCBSV are distinct virus species, with an additional probable new species (clade 2).

## Introduction

Cassava (*Manihot esculenta*) is a major staple food for more than 300 million people in sub‐Saharan Africa (FAO, 2013), including approximately 21 million people in Mozambique (Zacarias, 2008). However, its production is hampered by two viral diseases: cassava mosaic disease (CMD) and cassava brown streak disease (CBSD) (Thresh *et al*., 1997; Legg *et al*., 2006, 2011). CBSD causes yield losses of up to 70% in farmers’ fields in Africa, and economic losses of more than US$100 million annually (IITA, 2005). Symptoms of CBSD include vein clearing and leaf chlorosis, brown streaks on stems, and constrictions and necrosis in the roots of affected cassava plants (Storey & Nichols, 1936; Mbanzibwa *et al*., 2009a), making them unfit for consumption.

Cassava brown streak disease is caused by two positive‐sense single‐stranded (ss) RNA viruses (genus *Ipomovirus*, family *Potyviridae*): *Cassava brown streak virus* (CBSV) and *Uganda cassava brown streak virus* (UCBSV) (Mbanzibwa *et al*., 2009a; Winter *et al*., 2010; Ndunguru *et al*., 2015). However, UCBSV is not a single species, but seems to contain three species within the UCBSV species clade (Ndunguru *et al*., 2015). The size of viral genomes of CBSV and UCBSV are in the range of 8700–10 818 nucleotides (nt) and genomes are predicted to encode a polyprotein of 2902 amino acids (aa). These amino acids translate into 10 mature proteins: P1 (first potyviral protein), P3 (third potyviral protein), 6K1 (first 6‐kDa protein), CI (cylindrical inclusion), 6K2 (second 6‐kDa protein), VPg (viral genome‐linked protein), HAM1 (putative nucleoside triphosphate pyrophosphatase), NIa‐Pro (nuclear inclusion a–protease domain), NIb (nuclear inclusion b) and CP (coat protein). However, the HC‐Pro protein normally found in ipomoviruses is missing (Mbanzibwa *et al*., 2009a; Winter *et al*., 2010; Ndunguru *et al*., 2015). The proteins are involved in different functions. For example, the P1 protein was reported to play a significant role in virus replication (Pasin *et al*., 2014). The *HAM1* gene was initially identified in yeast in a mutation screen (Noskov *et al*., 1996) and it is also found across prokaryotes and eukaryotes (Galperin *et al*., 2006). Among viruses it was previously only observed for CBSV/UCBSV (Mbanzibwa *et al*., 2009b), but recently it was reported that *Euphorbia ringspot virus* (EuRSV) also encodes a *HAM1* protein with an uncharacterized function (Knierim *et al*., 2017).

The HAM1 in CBSV/UCBSV has conserved Maf/HAM1 motifs (Mbanzibwa *et al*., 2009a). The proteins with Maf/HAM1 domains have nucleoside triphosphate pyrophosphatase (NTPs) activities, which reduce mutation rates by preventing the incorporation of non‐canonical nucleotides into RNA and DNA (Galperin *et al*., 2006). The functions of HAM1 in CBSV and UCBSV are yet to be revealed but it was speculated to have a role in preventing excessive viral RNA mutation (Mbanzibwa *et al*., 2009a). Ogwok *et al*. (2014) suggested that HAM1 proteins might reduce mutation rates under oxidative stress conditions in mature cassava leaves, where CBSV viruses are found at the highest concentrations within the plant.

To understand the viruses causing CBSD in East Africa, there has been increased study of the genetic diversity of CBSVs, with deposition of at least 23 whole genome sequences (WGSs) in GenBank (Ndunguru *et al*., 2015; Alicai *et al*., 2016; Ateka *et al*., 2017). Ndunguru *et al*. (2015) reported increased diversity among the UCBSVs and suggested the possibility of new species. Alicai *et al*. (2016) produced the first coalescent based species tree estimation for CBSV and UCBSV that pointed to multiple species of both CBSV and UCBSV. The study also indicated that CBSV has a faster rate of evolution than UCBSV. Ateka *et al*. (2017) uncovered the aphid transmission‐associated DAG motif within the CP of all completely sequenced CBSV genomes at amino acid positions 52–54, but not in UCBSV. Upon further investigation, the DAG motif was also found at the same positions in the CP of two other ipomoviruses: *Squash vein yellowing virus* and *Coccinia mottle virus*.

In Mozambique, CBSD was first reported in 2002, where it was associated with CBSV (Thresh & Hillocks, 2003). In 2012, 1000 cassava leaf samples showing CBSD‐like symptoms were analysed using reverse transcription (RT)‐PCR and a set of primers (CBSDDR and CBSDDF2; Mbanzibwa *et al*., 2011a) that amplified a part of the *CP* gene, allowing researchers to screen for the species associated with CBSD. These results provided the first evidence for the occurrence of UCBSV in Mozambique (Amisse, 2013). Currently, there is only one WGS of CBSV (CBSV_MO_83_FN434436) from Mozambique in GenBank (Winter *et al*., 2010).

The limited availability of CBSV sequences from Mozambique makes it difficult to determine how genetically related the Mozambican isolates are to others reported in neighbouring countries in East and Central Africa. It also makes it difficult to anticipate the biological impacts on cassava crops, including symptom expression and root damage. Additional WGSs will allow assessment of the genetic diversity and evolution of the CBSV isolates in the country, and the design of appropriate tools for CBSD detection and diagnosis. The results reported in this study add to the body of knowledge on the genetic diversity and evolution of the CBSV isolates in Mozambique that is key to developing sustainable management strategies for this disease and increasing food security.

In this study, next‐generation sequencing was used to determine the WGSs of seven new CBSV isolates from cassava and two near full‐length CBSV genomes, one from cassava and another from a wild relative (*Manihot glaziovii*). All isolates were collected from major cassava‐growing areas in Mozambique. In addition, 26 WGSs reported from other countries were used to study the genetic diversity, recombination events, and best‐fit nucleotide substitution model among CBSV sequences from Mozambique.

## Materials and methods

### Field sample collection

A total of 30 leaves with CBSD symptoms were collected in northern (Nampula) and central (Zambezia) provinces in Mozambique in 2014. The samples were screened for the presence of CBSD‐associated viruses in the laboratory at the Mozambique Agricultural Research Institute (IIAM). Additionally, stem cuttings of plants with CBSD‐like symptoms were also collected and established in a screen house for further study. Field data were recorded as type of symptoms on leaves and roots, field geocoordinates, cultivar and sample number (Table 1).

**Table 1:** Geographic origin and cassava host cultivar name of the *Cassava brown streak virus* (CBSV) isolates collected in Mozambique and examined in this study

Isolate	District	Location	Altitude (m a.s.l.)	Cultivar	GenBank accession no.
CBSV_Mz_4	Namapa	13°59′36″S, 39°39′47″E	373	Ezalamalithi	KY563367
CBSV_Mz_5	Namapa	13°42′53″S, 39°47′28″E	311	Calamidade	KY563362
CBSV_Mz_8	Nampula	15°07′53″S, 39°23′57″E	252	Calamidade	KY563361
CBSV_Mz_16	Alto Molocue	16°22′11″S, 37°08′39″E	312	Bwana	KY563366
CBSV_Mz_20	Mocuba	17°06′09″S, 36°58′57″E	150	Cadri	KY563363
CBSV_Mz_22	Mocuba	17°35′18″S, 36°57′34″E	128	Robero	KY563364
CBSV_Mz_23	Quelimane	17°47′39″S, 36°54′16″E	31	Mulaleia	KY563365
CBSV_Mz_R1^a^	Namapa	13°46′58″S, 39°43′41″E	302	Mpopewe	—

a Isolate collected from *Manihot glaziovii*.

### RNA extraction and treatment for deep sequencing

Cassava leaves with CBSD symptoms from stems previously established in a screen house at IIAM in Nampula were collected for RNA extraction. Total RNA was extracted using the CTAB protocol (Lodhi *et al*., 1994; Xu *et al*., 2010) followed by DNase treatment and purification of RNA using a Direct‐Zol RNA Extraction kit (Zymo Research) following the manufacturer's instructions. RNA concentration and quality were determined using a NanoDrop and Qubit 2.0 (Invitrogen); both showed that all RNA samples were of good quality for library preparation and deep sequencing.

### RNA‐seq library preparation

Library preparation was done using a ScriptSeq v. 2 RNA‐Seq Library Preparation kit (Epicenter) following the manufacturer's instructions. The process consisted of removal of rRNA using a Ribo‐Zero kit process that removed >99% of cytoplasmic rRNA (and optionally, mitochondrial RNA) followed by RNA fragmentation and reverse transcription using random primers containing a 5′‐tagging sequence. The 5′‐tagged cDNA was then tagged at its 3′ end by the terminal‐tagging reaction to yield di‐tagged, single‐stranded cDNA. Following purification, di‐tagged cDNA was amplified by limited‐cycle PCR. This completed addition of the Illumina adaptor sequences and amplified the library for subsequent cluster generation. The amplified RNA‐Seq library was purified in preparation for cluster generation and 150‐bp paired‐end read sequencing on a MiSeq (Illumina). The process of library preparation and deep sequencing was conducted at the Agricultural Research Council in Pretoria, South Africa.

### *De novo* assembly and mapping

Raw reads were first trimmed using CLC genomics workbench v. 6.5 (clcgw) (CLC Bio) with the quality score limits set to 0.01, maximum number of ambiguities to 2 and any reads with <30 nt were removed. Contigs were then assembled using the *de novo* assembly function of clcgw with automatic word size, automatic bubble size, minimum contig length 350, mismatch cost 2, insertion cost 3, deletion cost 3, length fraction 0.5 and similarity fraction 0.9. The resulting contigs were subjected to a blast search using blastn and blastx (Altschul *et al*., 1990), to check which contigs matched viral sequences in GenBank. All contigs that matched positively (contigs of interest) to the reference available in GenBank were extracted and also imported into software geneious v. 9.0.4 (Biomatters Ltd). Mapping in geneious was performed with minimum overlap 10%, identity of 80% and an allowed gap of 10%. A consensus between the contig of interest from clcgw and the consensus from mapping in geneious was created in geneious by alignment with mafft (Katoh *et al*., 2002). A custom read blast database was created in clcgw to finalize the ambiguities and to help generate the final sequence. All annotations and edits were made using geneious.

### Genome alignment

A total of 26 full genome reference sequences previously published, comprising 12 CBSV and 14 UCBSV, were downloaded from GenBank and imported into geneious v. 9.0 (Kearse *et al*., 2012). These 26 sequences, in addition to the seven new WGSs from Mozambique and the two near full‐length genomes, were aligned in geneious using the mauve plugin. Nucleotide alignments were translated into proteins using the mafft translate align option available in geneious followed by visual verification.

### Recombination analysis

Recombination detection analysis for CBSV and UCBSV sequences in this study was done using RDP v. 4.63 (RDP Beta 4.63) (Martin *et al*., 2015). The previously saved fasta file containing 33 aligned sequences of CBSV and UCBSV was imported into RDP4. The detection methods used were 3seq, bootscan, chimaera, genecov, lard, maxchi, rdp and siscan implemented in the RDP4 package with parameters set to default settings. The recombination events were computed with a highest acceptable *P*‐value of 0.05. An event was accepted if detected by three or more of the programs used.

### Phylogenetic analysis

#### Gene trees

Nucleotide alignments of CBSV sequences from this study were included with full‐length genomes of 12 CBSV (11 from Tanzania and an older one from Mozambique) and 14 UCBSV isolates from East African countries available in GenBank. To determine the best fitting model of molecular evolution, jmodeltest (Darriba *et al*., 2012) was run on the final dataset and GTR+I+G was used to carry out the mrbayes v. 3.3.2 (Ronquist *et al*., 2012) analyses. mrbayes v. 3.2.2 (Ronquist & Huelsenbeck, 2003) phylogenetic analysis was run in parallel (four processors) on the Magnus supercomputer (Pawsey Supercomputer Centre, Perth, Western Australia). The analysis was run for 30 million generations and trees were sampled every 1000 generations. All runs reached a plateau in likelihood score (i.e. stationarity), which was indicated by the standard deviation of split frequencies (0.0015), and the potential scale reduction factor (PSRF) was close to 1, indicating the MCMC chains converged. Convergence of the runs was also checked using tracer v. 1.6 and the effective sample size (ESS) values were well above 200 for each run.

#### Whole genome phylogenetic analysis

Phylogenetic analyses of the whole genome nucleotide as well as the deduced amino acid sequences were conducted with exabayes v. 1.4.1 (Aberer *et al*., 2014) as described in Ndunguru *et al*. (2015) under GTR+I+G model. exabayes was run in parallel across 384 nodes on the Magnus supercomputer. Analyses were run for 1 million generations with sampling every 500 generations. Each analysis consisted of four independent runs, each using four coupled Markov chains. The run convergence was monitored by finding the plateau in the likelihood scores (standard deviation of split frequencies <0.0015). The first 25% of each run was discarded as burn‐in for the estimation of a majority rule consensus topology and posterior probability for each node. Additionally, the evolutionary distances over sequence pairs between different groups (clades) were calculated using mega 6 software under the maximum composite likelihood model (Tamura *et al*., 2013).

### Species delimitation

Species delimitation was assessed using the standard Kimura two‐parameter (K2P) interspecies distance plus two more stringent measures of taxon distinctiveness, as described in Rosenberg's reciprocal monophyly P(AB) (Rosenberg, 2007) and Rodrigo's P(RD) (Rodrigo *et al*., 2008). The species delimitation plugin (Masters *et al*., 2010) for geneious (Kearse *et al*., 2012) was used to calculate P(AB) and P(RD). Species delimitation was assessed using the exabayes (Aberer *et al*., 2014) tree generated from the WGS. The tip‐to‐root process is designed to delimit species because the species delimitation measures dictate where to draw the species line.

## Results

### CBSD symptoms in the field

**Figure 1:**
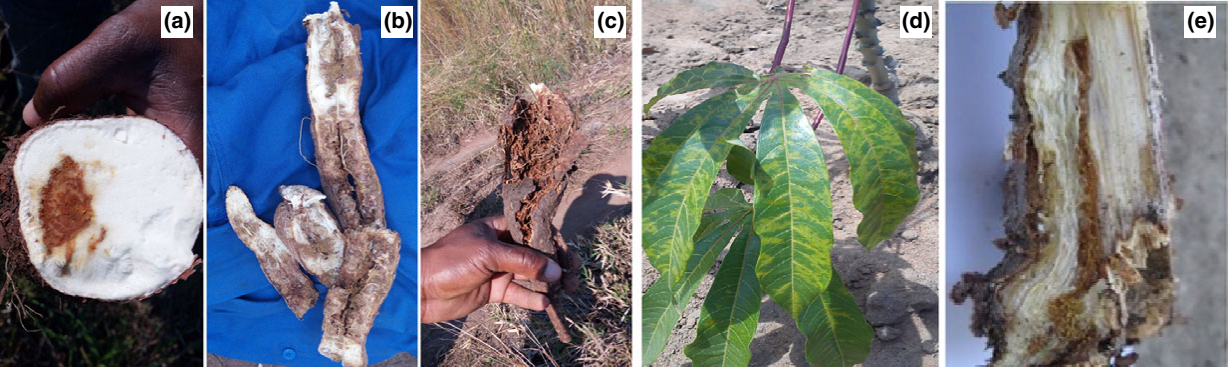
Typical CBSD symptoms observed in the cassava farmers’ fields during sampling in Nampula and Zambezia provinces in Mozambique. (a) Moderate root necrosis, (b) severe root necrosis, (c) highly severe root necrosis and rot, (d) leaf chlorosis, and (e) inner necrosis of stems along the xylem.

Cassava plants showed typical CBSD symptoms including: chlorosis on leaves and necrosis on stems and roots. All plants with foliar symptoms (Fig. 1d) showed clear necrosis on roots when uprooted (Fig. 1a–c). In addition, when stems of plants with foliar symptoms were dissected longitudinally, uncommon symptoms of brown necrosis were observed along the xylem tissue (Fig. 1e).

### WGSs for CBSV isolates in Mozambique

Before this study, only one WGS of CBSV from Mozambique was available in GenBank. In the present study, seven new WGSs of CBSV were generated, as well as two near full‐length sequences from Mozambique. One CBSV isolate was obtained from a cassava relative, *M. glaziovii*, and the rest from cassava cultivars. The sequence lengths of the CBSV isolates were in the range of 8778–9047 nucleotides (nt) (Table 2).

**Table 2:** Next‐generation sequencing data and genome size of the seven *Cassava brown streak virus* (CBSV) isolates collected in Mozambique and used in this study

Sample	No. of reads obtained	No. of reads after trimming	No. of contigs produced^a^	Contig length mapped to the consensus (nt)	Contig length (nt)	Average coverage (fold)^b^	No. of reads mapped to contig of interest	CBSV ref. seq. used for mapping	Length of consensus sequence from mapping (nt)^c^	No. of reads mapped to ref. seq.	Average coverage (fold)^c^	Final sequence length (nt)
CBSV_Mz_4	2 460 222	2 406 924	3709	230, 823, 2235	4751, 3683, 617	26, 13, 13	1290	TZ 19‐1	8778	1290	22	8778
CBSV_Mz_5	3 608 398	3 562 380	4081	179	9003	163	9736	TZ GQ329864	9003	9796	165	9003
CBSV_Mz_8	1 541 978	1 519 424	2750	110, 227, 313	6044, 1610, 1177	109, 69, 61	6052	TZ MAF 49	9047	5807	96	9047
CBSV_Mz_16	2 591 794	2 563 714	2854	278, 655, 1026	1224, 2186, 1898	17, 21, 8	1071	TZ MAF 49	8895	1071	17	8895
CBSV_Mz_20	2 872 090	2 826 912	5871	61, 42	1525, 7257	815, 819	54 019	TZ MAF 49	9028	54 019	848	9028
CBSV_Mz_22	6 992 534	6 953 496	10 791	215, 3261	8732, 1304	293, 11	17 500	TZ MAF 49	9007	17 500	296	9007
CBSV_Mz_23	2 635 892	2 619 932	1982	3	9014	65	4259	TZ MAF 49	9014	4259	66	9014

a clc.

b clcgw.

c geneious.

### Phylogenetic analysis of whole genomes and individual genes of CBSV and UCBSV

**Figure 2:**
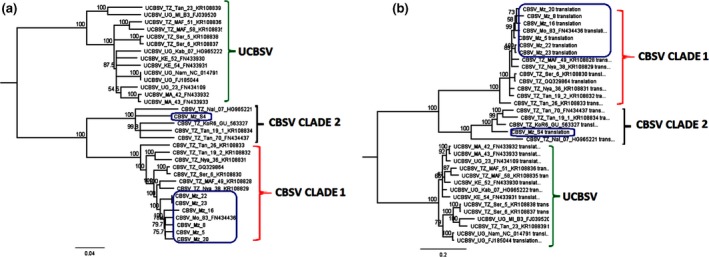
Phylogenetic trees based on (a) nucleotides and (b) deduced amino acids of full‐length sequences of the seven new Mozambican CBSV isolates sequenced in this study (indicated by the blue rectangle) as well as 12 CBSV and 14 UCBSV isolates previously reported in East Africa. Analyses were run for one million generations with sampling every 500 generations. The number at each branch represents the bootstrap value. The scale bar represents (a) nucleotide and (b) amino acid substitutions per site.

Phylogenetic analyses with nucleotides and amino acids (aa) of WGSs revealed the existence of three major groups: UCBSV and two distinct clades or groups of CBSV sequences. The analysis grouped the seven new CBSV sequences from Mozambique into two clades: clades 1 and 2 (Fig. 2).

Clade 1 comprised most CBSV sequences. Six out of seven of the new Mozambique sequences were clustered in clade 1 together with the majority of CBSV sequences from Tanzania. In contrast, CBSV clade 2 comprised a minority of sequences, of which only one was from Mozambique. Interestingly, among the CBSV sequences in clade 1, those from Mozambique clustered distinctly from sequences reported previously from Tanzania (Fig. 2). A near full‐length genome of a CBSV isolate from *M. glaziovii* clustered within CBSV clade 1 (results not shown).

**Figure 3:**
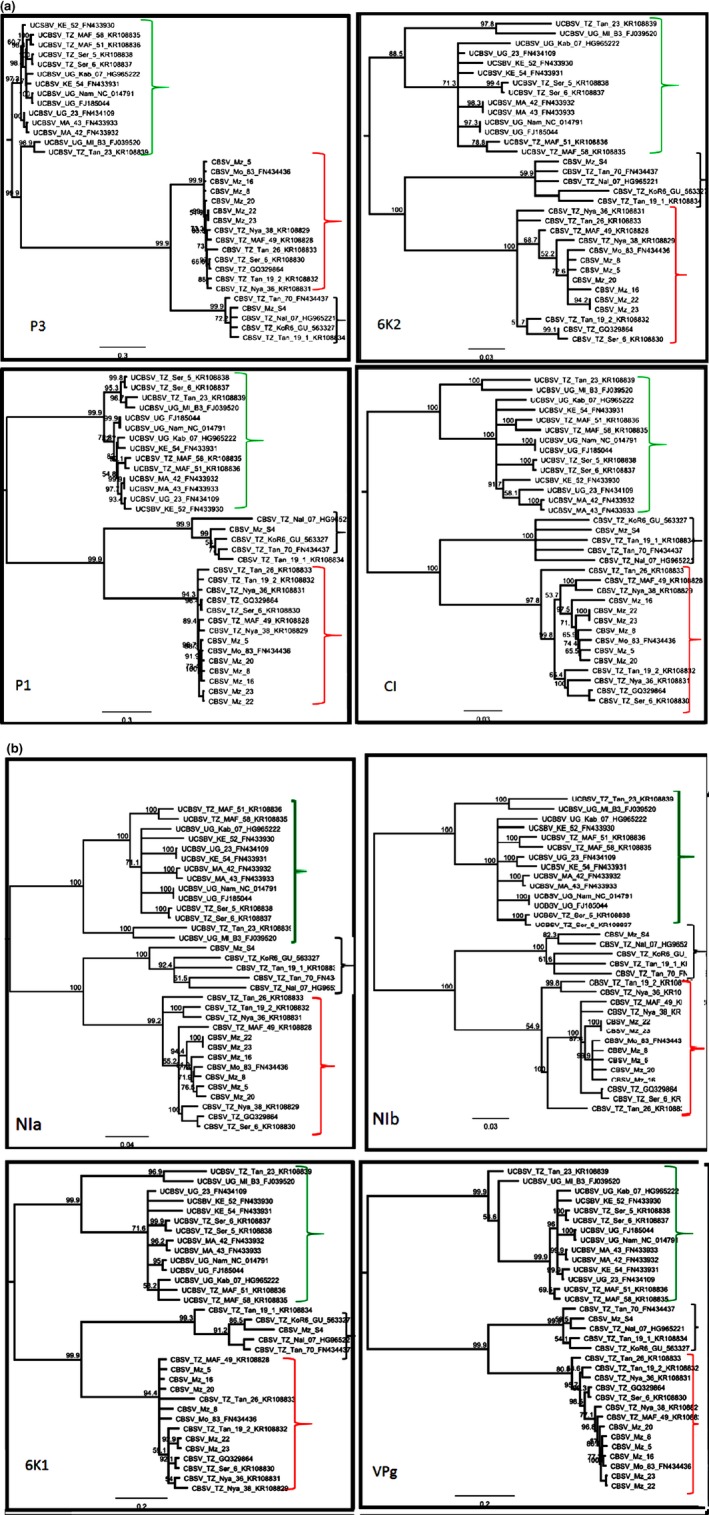
Phylogenetic trees based on the individual gene nucleotide sequences of P3, 6K1, P1, CI, NIa, NIb, 6K2 and VPg of CBSD‐associated viruses (CBSV and UCBSV) previously reported in other countries and CBSV isolates collected in Mozambique for this study. The tree topology was used to compare and analyse the evolution of different genomic regions within CBSD‐associated viruses. Trees for eight out of 10 genes showed the same topology and placed all isolates into three clades: red brackets represent CBSV clade 1, black brackets represent CBSV clade 2, in which only one isolate from Mozambique clustered; the green brackets include all UCBSV isolates. The trees were generated using best‐fit model pre‐selected in jmodeltest. The number at each branch represents the bootstrap value (1000 replicates). The scale bar represents nucleotide substitutions per site.

**Figure 4:**
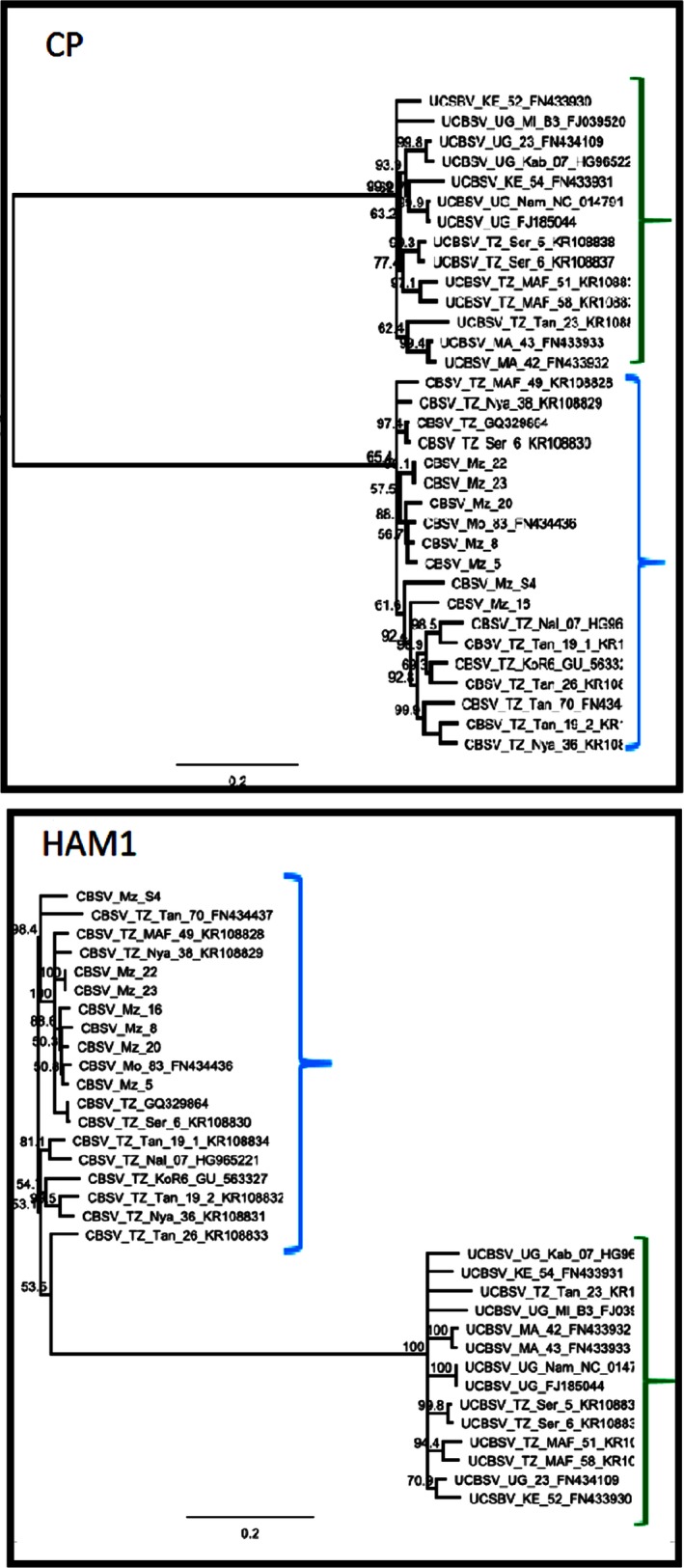
Phylogenetic trees based on the *CP* and *HAM1* gene nucleotide sequences of CBSD‐associated viruses (CBSV and UCBSV). In contrast to other genes, the *HAM1* and *CP* gene sequences placed all isolates in two distinct clades: one clade of UCBSV (indicated by green brackets) and a second clade comprising all CBSV sequences (clades 1 and 2, indicated by blue brackets). The trees were generated using best‐fit model preselected in jmodeltest. The number at each branch represents the bootstrap value (1000 replicates) and the scale bar represents nucleotide substitutions per site.

To determine how well the trees (generated using nt and aa) of WGSs reflected the individual gene trees, the tree topologies of WGSs and individual genes were compared. Phylogenetic analyses with eight of the 10 CBSV/UCBSV genes generated the same tree topologies as the WGSs, showing three distinct groups: UCBSV, CBSV clade 1 and CBSV clade 2 (Fig. 3). In contrast, phylogenetic analysis for two genes (*HAM1* and *CP*) did not distinguish the three distinct groups, but only two major groups: UCBSV and CBSV sequences (Fig. 4).

Pairwise comparison between the seven new CBSV complete nt and aa sequences revealed sequence identities of 79.3–100% (Table S1) and 86.7–100%, respectively (Table S2). Further comparisons between the new CBSV sequences and an older published sequence (CBSV_MO_83_FN434436) from Mozambique revealed sequence identities of 79.3–98% (Table S1) and 86.7–98.8% (Table S2) for nt and aa sequences, respectively.

Full genome sequences of clades 1 and 2 shared nt identities of 79.0–80.4% (Table S1) and deduced aa identities of 86.5–88.2% (Table S2). Group mean distance between clades 1 and 2 was 30% (data not shown). Within clade 1, isolates shared high identities of 91.8–100% nt (Table S1) and deduced aa identities of 95.4–100% (Table S2); whereas within CBSV clade 2, sequences shared nt identities of 89.4–91.4% (Table S1) and deduced aa identities of 92.8–95.3% (Table S2).

Clades 1 and 2 were highly divergent to all UCBSV sequences: clade 1 shared identity of just 69.1–70.2% nt (Table S1) and 73.3–74.5% aa (Table S2) with all UCBSV sequences; similarly, clade 2 shared identity of 68.5–70.1% nt (Table S1) and 72.9–74.7% aa (Table S2). When the nt identity based on *HAM1* sequences was compared, higher identity (91.3–100%) was observed between CBSV sequences from Mozambique than others from Tanzania (86.4–99.7%; Table S3).

### Comparison of nucleotide and amino acid sequences of CBSV clades 1 and 2

**Figure 5:**
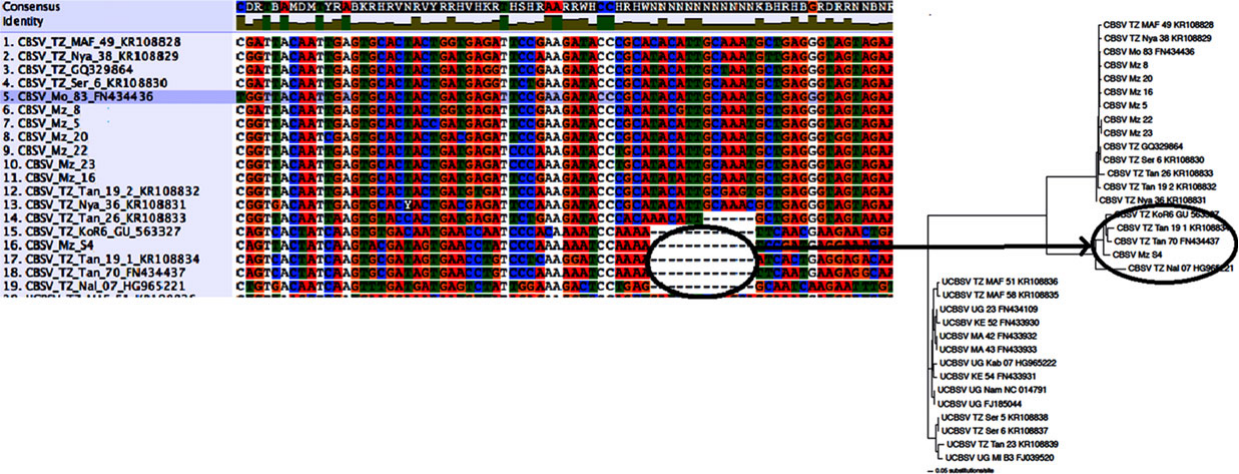
Alignment of *P1* nucleotide sequences of CBSV isolates. Isolates of clade 2 were characterized by a deletion of 12 nucleotides; this specific deletion was exclusive for all isolates of CBSV clade 2 (indicated by ellipse in the phylogenetic tree) and was not observed for isolates of clade 1 or UCBSV.

Nucleotides and amino acids were aligned and analysed for WGSs to detect regions that differed most between CBSV clades 1 and 2. Interestingly, all sequences belonging to clade 2 lacked 12 nt, corresponding to four amino acids within the *P1* gene, compared to sequences of CBSV clade 1 or UCBSV (Fig. 5).

Divergences of amino acid residues between CBSV genomes of isolates belonging to clades 1 and 2 were observed. Most proteins displayed amino acid residues that were specific to either clade (Fig. S1). Among all CBSV proteins, the P1 protein had the highest divergence of amino acid residues between clades 1 and 2 (Fig. S1). The CP had the lowest divergence (Fig. S2), with similar observations for HAM1 (data not shown). A small difference in amino acid residues in the CP was observed at position 7880–8030 in the amino acid alignment of the polyprotein; however, no amino acids were specific for either clade (Fig. S2).

### Recombination analysis

**Figure 6:**
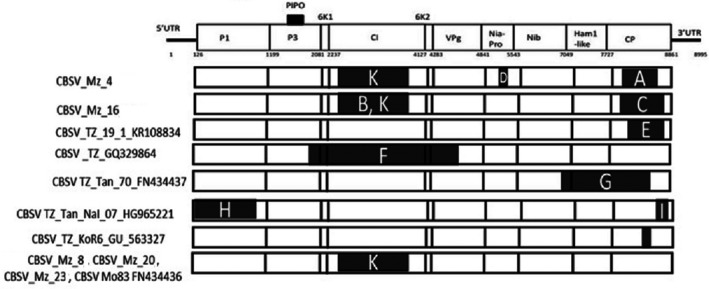
Recombination map of CBSV genome. Analysis of possible recombination in full‐length genomes of CBSD‐associated viruses was done using RDP Beta 4.63. Eleven recombination events (represented by uppercase letters) were observed among 19 CBSV isolates, with five of the 11 events in CBSV isolates from Mozambique. Event K in the *CI* gene only occurred in CBSV isolates from Mozambique, and the major and minor parents were isolates from Tanzania: CBSV_TZ_MAF_49 and CBSV_TZ_GQ329864.

Using RDP4, a recombination analysis was performed for the seven CBSV sequences from Mozambique, as well as 12 CBSV and 14 UCBSV sequences previously determined. Among the sequences, 11 recombination events were detected in the CBSV sequences and three (data not shown) in the UCBSV sequences. At least one or more recombination events were observed for each individual CBSV gene. Across the gene sequences, the most recombination events (five) were observed in the *CP* gene followed by *CI* (Fig. 6). Of the 11 events detected in CBSV sequences, five were observed in the CBSV sequences from Mozambique: two were detected in *CI*, one in *NIa* and two in *CP*. Events A, B, H and I were supported by six methods and were observed in isolates CBSV_Mz_4, CBSV_Mz_16 and TZ_Tan_NaI_07_HG965221 (Table 3).

**Table 3:** Summary of recombination events identified by RDP v. 4.63 (Martin *et al*., 2015) in the whole genome sequence of *Cassava brown streak virus* (CBSV) isolates

Event	Recombinant	Parent		Detection methods^a^	*P*‐value	Break points^b^	
		Major	Minor			Begin	End
A	CBSV_Mz_4	Unknown	CBSV_Mo83_FN434436	B, C, G, M, **R**, S	4.0 × 10^−33^	7962	8629
B	CBSV_Mz_16	CBSV_Mz_8	CBSV_Mz_4	B, C, G, M, **R**, S	4.5 × 10^−32^	2680	2864
C	CBSV_Mz_16	CBSV_Mo83_FN434436	Unknown	B, C, M, **R**, S	7.7 × 10^−27^	7946	8686
D	CBSV_Mz_4	CBSV_Tan70_FN434470	CBSV_Mo83_FN434436	B, **G**, M, R	4.1 × 10^−13^	5325	5426
E	CBSV_TZ_19_1_KR108834	Unknown	CBSV_Mz_20	B, C, M, **R**	1.1 × 10^−11^	7960	8681
F	CBSV_TZ_GQ329864	CBSV_TZ_Nya_38	CBSV_TZ_Tan19_2_KR108832	**C**, L, M, S	4.5 × 10^−10^	2021	4468
G	CBSV_TZ_Tan_70_FN434437	CBSV_TZ_19_1_KR108834	Unknown	**B**, C, M, R, S	3.8 × 10^−15^	6863	8506
H	CBSV_TZ_Tan_NaI_07_HG965221	CBSV_Mz_4	Unknown	B, C, G, M, R, **S**	3.8 × 10^−21^	1	717
I	CBSV_TZ_Tan_NaI_07_HG965221	CBSV_Mz_4	Unknown	B, C, G, M, R, **S**	3.8 × 10^−21^	8630	8748
J	CBSV_TZ_KoR6_GU_563327	Unknown	CBSV_TZ_Nya_36_KR108831	B, C, M, **R**	4.9 × 10^−8^	7861	8538
K	CBSV_Mz_4, CBSV_Mz_8, CBSV_Mz_16, CBSV_Mz_20, CBSV_Mz_23, CBSV Mo83 FN434436, CBSV_Mz_5	CBSV_TZ_MAF_49	CBSV_TZ_GQ329864	**3**, C, M, R	2.4 × 10^−6^	2384	3274

a 3, 3seq; B, bootscan; C, chimaera; G, genecov; L, lard; M, maxchi; R, rdp; S, siscan. The methods whose *P*‐values are shown are indicated in bold and underlined.

b The breakpoint positions of recombination are indicated in relation to the nucleotide sequence of the whole genome sequence of CBSV isolates.

The highest number (three) of recombination events was detected in isolates CBSV_Mz_4 and CBSV_Mz_16, but most isolates had only one or two events. Interestingly, event K was exclusive to CBSV isolates from Mozambique and did not occur in isolates from Tanzania. The major and minor parents of this recombination event (K) were CBSV_TZ_MAF_49 and CBSV_TZ_GQ329864, both from Tanzania (Fig. 6). This is the first comprehensive study to provide evidence of recombination events in the species associated with CBSD in southern Africa.

No recombination event was observed between CBSV and UCBSV, or within sequences of CBSV clade 2; however, there were recombination events between CBSV clades 1 and 2, and among CBSV sequences of clade 1.

### Species delimitation

The species delimitation was based on three species delimitation statistics: K2P interspecies distance plus two more stringent measures of taxon distinctiveness, P(AB) and P(RD). These reconfirmed that CBSV and UCBSV were distinct species. A probable additional species/clade among CBSV isolates was also observed (Table 4).

**Table 4:** Species delimitation for *Cassava brown streak virus* generated on exabayes tree using whole genome nucleotides

Species	Closest species	Monophyletic?	Intra Dist^a^	Inter Dist^b^	Intra:Inter^c^	P ID (strict)^d^	P ID (liberal)^e^	Av(MRCA‐tips)^f^	P(RD)^g^	P(AB)^h^
1	2	Yes	0.074	0.896	0.08	0.88 (0.75, 1.0)	0.97 (0.87, 1.0)	0.1518	0.05	4.9 × 10^−6^
2	1	Yes	0.029	0.896	0.03	0.98 (0.93, 1.0)	1.00 (0.97, 1.0)	0.1400	0.05	4.9 × 10^−6^

a Average pairwise tree distance among members of a predefined clade.

b Average pairwise tree distance between members of the group of interest and its sister taxa (K2P distance).

c The ratio of Intra Dist to Inter Dist.

d Mean probability, with a 95% confidence interval for a prediction of making a correct identification of an unknown specimen being found only in the group of interest.

e Mean probability, with a 95% confidence interval for a prediction of making a correct identification of an unknown specimen being sister to or within the group of interest.

f Mean distance between the most recent common ancestor of the species and its members.

g Rodrigo's P(RD), probability that a clade has the observed degree of distinctiveness.

h Rosenberg's reciprocal monophyly.

## Discussion

Phylogenetic analysis of the seven new CBSV WGSs from Mozambique obtained in the present study, as well as those published from other countries, allowed a more comprehensive analysis than was previously possible, as there was only one WGS from Mozambique before this study. This analysis supports the existence of two clades among CBSV sequences, and for the first time shows a 12‐nt deletion in the *P1* gene corresponding to four amino acids in sequences of clade 2, whereas no deletion was observed in sequences of clade 1. CBSV clades 1 and 2 were genetically distinguishable from UCBSV isolates reported in East Africa (Mbanzibwa *et al*., 2009a,b, 2011a; Winter *et al*., 2010) and Mozambique (Amisse, 2013). These results suggest that, in Mozambique, CBSD is caused by more than two CBSD‐associated virus species (UCBSV and two species of CBSV), rather than only two as previously thought.

The two clades of CBSV have been previously reported, based on the *P1* gene sequences (Mbewe *et al*., 2017) and WGSs (Alicai *et al*., 2016). This study presents molecular evidence that among the 10 gene sequences of CBSV, eight can be used to discriminate the two clades. The findings are well supported by tree topologies across eight gene sequences that consistently showed two clades among CBSV sequences, in contrast to *HAM1* and *CP* which joined the two clades as one. The results further show that primers based on *HAM1* and *CP* sequences may not distinguish isolates from different clades of CBSV. However, primers based on *HAM1* and *CP* may provide a very robust tool for general screening of CBSV for breeders, when there is no need to distinguish the strains or variants within CBSV clades. It was observed that *HAM1* and *CP* were the most conserved genes between CBSV clades 1 and 2, in contrast to *P1*, which was the most variable gene, consistent with the observations of Mbewe *et al*. (2017). The high conservation of *HAM1* and *CP* among isolates of CBSV clades 1 and 2 observed here suggests that both were maintained during speciation within CBSV.

Previous studies have observed different biological reactions in terms of symptom severity in *Nicotiana benthamiana* between infections using CBSV and UCBSV (Winter *et al*., 2010). In this study, significant variation was observed in protein sequences of each clade, with some specific amino acids appearing at the same position in most of the coding regions, which could suggest different biological functions. Further studies should determine differences in biological functions in the cassava host. It is speculated that some released cassava varieties will have different levels of tolerance based on which CBSV clade‐type viruses they were originally screened with (a fact that may not even be possible to know). Future infection assays to screen the tolerance/resistance of released varieties against the two CBSV clades isolates will be required. This will ensure that appropriate cassava varieties are deployed in locations where a specific strain or clade occurs.

Recombination was detected in the seven new sequences in this study. Similar results were previously observed by Winter *et al*. (2010) and Mbanzibwa *et al*. (2011b) based on one WGS from Mozambique. The present study adds strong evidence for recombination between sequences of CBSV from Southern and Eastern Africa, with most recombination events occurring in *CP* followed by *CI*. Ndunguru *et al*. (2015) and Mbanzibwa *et al*. (2011b) have previously carried out recombination detection analysis with CBSV sequences from East Africa and observed similar results, with most recombination events detected in the *CP* as observed in this study. However, whereas in the present study *CI* was the genomic region with the second most recombination events among CBSV sequences, Ndunguru *et al*. (2015) and Mbanzibwa *et al*. (2011b) found *HAM1* to be the gene with the second most recombination events. Interestingly, event ‘K’ in the *CI* gene was exclusive to CBSV sequences from Mozambique, whose two CBSV parents were from Tanzania. No recombination was observed between UCBSV and CBSV, which is consistent with previous studies (Mbanzibwa *et al*., 2011b; Ndunguru *et al*., 2015).

The 4‐aa deletion observed in the P1 protein shows that CBSV sequences in clades 1 and 2 can be discriminated based on the amino acid deletions. The P1 protein is multifunctional, responsible for adaptation of the potyviruses to a wide range of host species (Valli *et al*., 2007) and binds ssRNA (Brantley & Hunt, 1993). A specific domain (RSSRAMKQKRARERRRAQQ) of the P1 protein was observed in *Turnip mosaic virus* that potentially interacts with nucleic acids (Soumounou & Laliberté, 1994) and in CBSV, the P1 functions as a suppressor of RNA silencing (Mbanzibwa *et al*., 2009a). CBSV and UCBSV might use binding through a ‘bridge’ formed by the virus‐encoded P1 protein with putative receptors located in the whitefly maxillary stylet (Dombrovsky *et al*., 2014). Thus, it is possible that this deletion may affect the transmission efficiency of the virus by whiteflies – a finding that requires extensive further research. The P1 protein also plays a significant role in virus replication (Pasin *et al*., 2014). The mutations in P1 may affect replication of the virus in the host and could also affect virus epidemiology and virulence.

In two previous studies, a short 344‐nt sequence of CBSV has been obtained from a cassava relative, *M. glaziovii* (Mbanzibwa *et al*., 2011a; Amisse *et al*., 2019). However, it was not known how the CBSV sequence collected in *M. glaziovii* was genetically related to isolates from cassava. This study provided the first near full‐length (8024 nt) sequence of CBSV from *M. glaziovii* showing high similarity (96.1–100%) with the CBSV sequences from cassava cultivars.

Analyses to determine speciation were carried out by Ndunguru *et al*. (2015), where support was found for dividing UCBSV into additional species, but not CBSV. Several comprehensive evolutionary models and statistical programs were used here to confirm that CBSV and UCBSV are distinct virus species. A criterion based on distance (percentage similarity) and another based on tree topology confirmed CBSV and UCBSV as distinct species, as previously reported (Mbanzibwa *et al*., 2009b; Ndunguru *et al*., 2015) and supported the existence of two species among CBSV clade 1 and 2 sequences.

Nucleotide and amino acid identities between CBSV clades 1 and 2 WGSs were in the range of 79.1–80.4% and 86.5–88.2%, respectively, which does not meet species delimitation criteria based on use of a priori genetic distance threshold as the cut off (<77% nt sequence and <82.9% aa sequence identity of the whole genome) (Adams *et al*., 2005; ICTV, 2005, 2011), while the other species delimitation criteria (reciprocal monophyly) used in this study indicate an additional species within the CBSV clade. In situations where the existence of two clades/species among CBSV sequences is confirmed, with one clade exhibiting substantial genetic variability from the other, as shown here, but the percentage identity criteria is not satisfied, the elevation of these two clades as different strains and/or species requires further research and discussion. However, this study further suggests that there are probably two species among the CBSV isolates in Mozambique. This is key knowledge that will advise the development of sustainable management strategies for CBSD to ensure food security.

## Supporting information


**Figure S1.** Alignment of the deduced amino acid sequences of *P1* showing divergence of amino acid residues in some positions. Amino acid residue divergence between CBSV clade 1 (comprising most CBSV Mozambique isolates) and CBSV clade 2 was observed, and the occurrence of specific residues in some positions were specifically related to specific clades. The amino acid residues not shared between the two clades are shaded in different colours.

**Figure S2.** Alignment of the deduced amino acid sequences in the coat protein CP showing high consensus that is unlike a high divergence observed in the other genes.

**Table S1.** Pairwise comparison of the full‐length genome nucleotide sequences of virus isolates expressed as percentage nucleotide similarity between CBSV isolates from cassava samples from Mozambique (bold) and other countries as calculated by clustalW algorithm.

**Table S2.** A pairwise comparison of the deduced amino acid identity for polyprotein of virus isolates expressed as percentage amino acids identity between CBSV isolates from cassava samples from Mozambique (bold) and other countries as calculated by clustalW algorithm.

**Table S3.** Nucleotide sequence identity (%) of HAM1 protein of CBSV isolates. The identity values in bold represent those shared between CBSV isolates from Mozambique, while the values not in bold represent the identity values shared between isolates previously reported elsewhere.
