# Supplementary figures and images for: Analyses of seven new whole genome sequences of cassava brown streak viruses in Mozambique reveals two distinct clades: evidence for new species
Source: Plant Pathol. 2019 Mar 10;68(5):1007–18. doi: 10.1111/ppa.13001 (PMC6563196; doi:10.1111/ppa.13001)

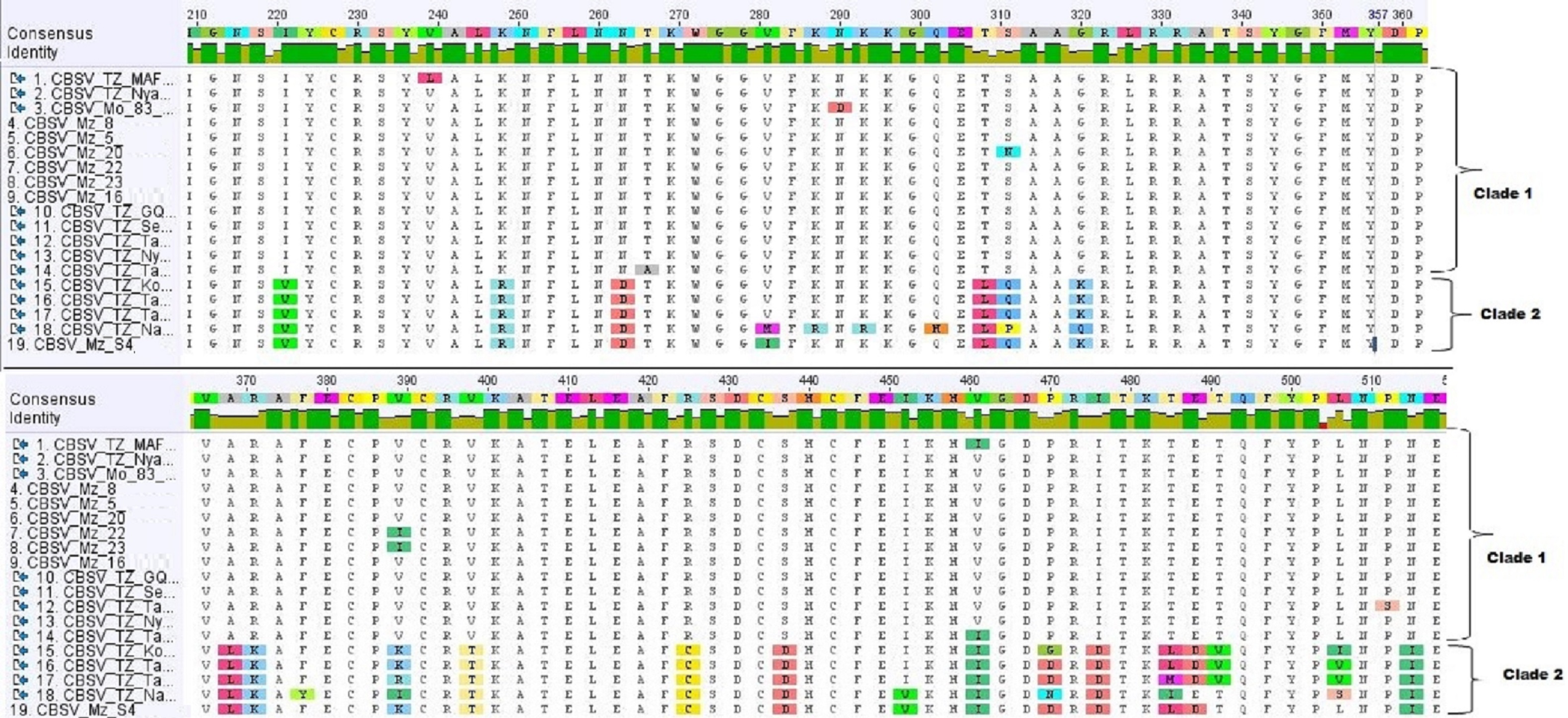

Supplement: Supplementary file 1 — Figure S1. Alignment of the deduced amino acid sequences of P1 showing divergence of amino acid residues in some positions. Amino acid residue divergence between CBSV clade 1 (comprising most CBSV Mozambique isolates) and CBSV clade 2 was observed, and the occurrence of specific residues in some positions were specifically related to specific clades. The amino acid residues not shared between the two clades are shaded in different colours. [file PPA-68-1007-s001.jpg]

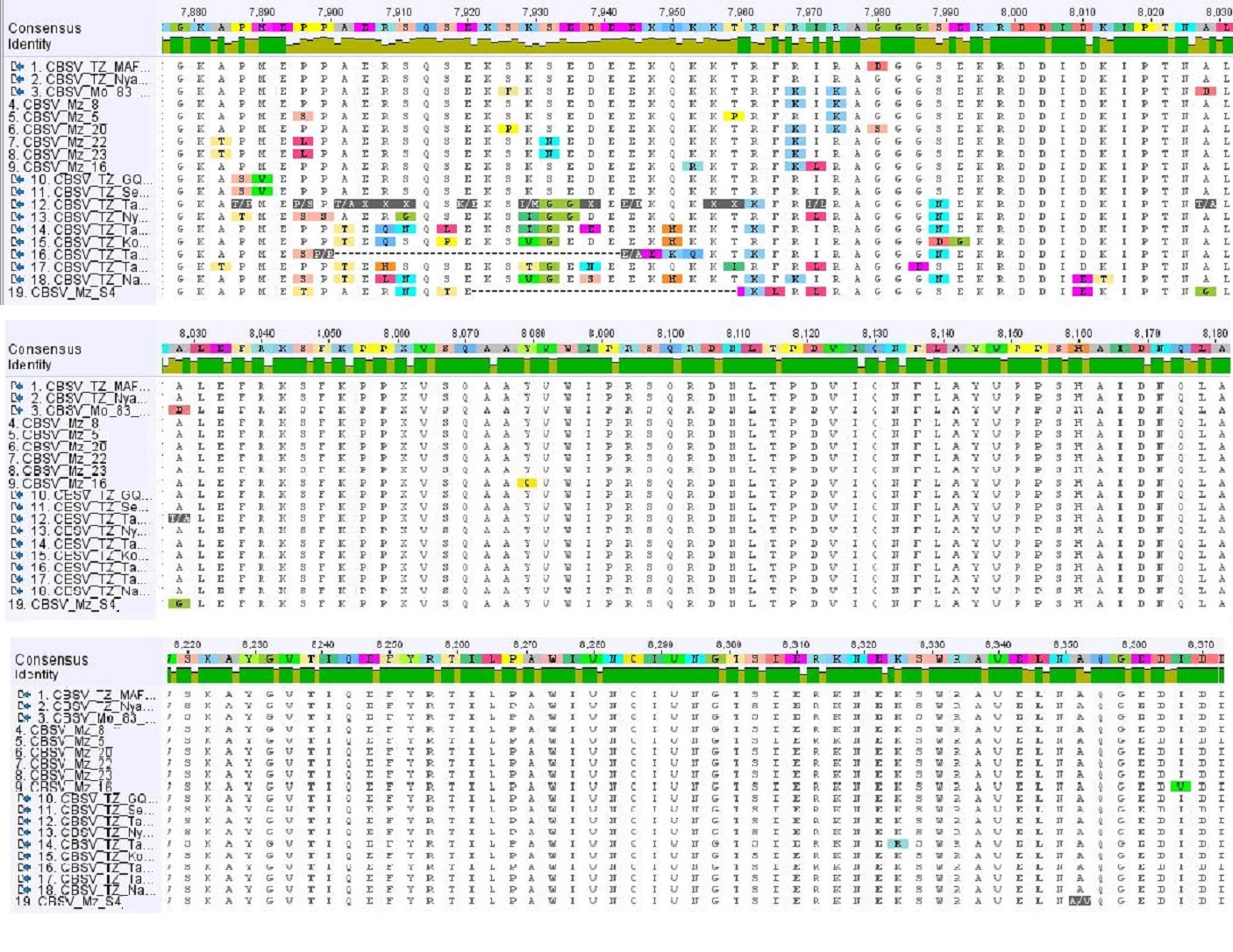

Supplement: Supplementary file 2 — Figure S2. Alignment of the deduced amino acid sequences in the coat protein CP showing high consensus that is unlike a high divergence observed in the other genes. [file PPA-68-1007-s002.JPG]
